# Characterization of Gastric Mucosa Biopsies Reveals Alterations in Huntington's Disease

**DOI:** 10.1371/currents.hd.858b4cc7f235df068387e9c20c436a79

**Published:** 2015-06-26

**Authors:** Andrew C McCourt, Kirsty L O'Donovan, Eva Ekblad, Elin Sand, David Craufurd, Anne Rosser, David Sanders, Nicholas Stoy, Hugh Rickards, Nils Wierup, Gillian P. Bates, Maria Björkqvist, Oliver Quarrell

**Affiliations:** Department of Experimental Medical Sciences, Brain Disease Biomarker Unit, Wallenberg Neuroscience Center, Lund University, Lund, Sweden; Department of Clinical Genetics, Sheffield Children's Hospital, Sheffield Children's NHS Foundation Trust, Sheffield. South Yorkshire, UK; Department of Experimental Medical Science, Lund University, Lund, Sweden; Department of Neurogastroenterology, Experimental Medical Science, Lund University, Lund, Sweden; University of Manchester, Manchester Academic Health Sciences Centre and Central Manchester University Hospitals NHS Foundation Trust, Manchester, UK; School of Biosciences and Medicine, Cardiff University, Cardiff, Wakes, UK; Department of Gastroenterology, Royal Hallamshire Hospital, Sheffield, South Yorkshire, UK; Department of Systems Biologym University of Surrey, Guildford, Surrey, UK; Department of Neuropsychiatry, University of Birmingham, Birmingham, West Midlands, UK; Lund University Diabetes Centre, Lund University, Malmö, Sweden; Department of Medical and Molecular Genetics, Kings College London, London, UK; Department of Experimental Medical Science, Wallenberg Neuroscience Center, Lund University, Lund, Sweden; Department of Clinical Genetics, Sheffield Children's Hospital, Sheffield Children's NHS foundation Trust, Sheffield, South Yorkshire, UK

## Abstract

Weight loss is an important complication of Huntington’s disease (HD), however the mechanism for weight loss in HD is not entirely understood. Mutant huntingtin is expressed in the gastrointestinal (GI) tract and, in HD mice, mutant huntingtin inclusions are found within the enteric nervous system along the GI tract. A reduction of neuropeptides, decreased mucosal thickness and villus length, as well as gut motility impairment, have also been shown in HD mice. We therefore set out to study gastric mucosa of patients with HD, looking for abnormalities of mucosal cells using immunohistochemistry. In order to investigate possible histological differences related to gastric acid production, we evaluated the cell density of acid producing parietal cells, as well as gastrin producing cells (the endocrine cell controlling parietal cell function). In addition, we looked at chief cells and somatostatin-containing cells. In gastric mucosa from HD subjects, compared to control subject biopsies, a reduced expression of gastrin (a marker of G cells) was found. This is in line with previous HD mouse studies showing reduction of GI tract neuropeptides.

## Introduction

Most studies into the pathology of Huntington’s disease (HD) focus on the basal ganglia and cerebral cortex^1^. However, mutant huntingtin is expressed throughout the body and abnormalities have been noted in peripheral tissues, not considered secondary to neuronal damage^2^^,^ 3 ^,^ 4.

Weight loss is one of the most common peripheral features of HD^5^^,^ 6. The underlying mechanisms are not, however, entirely known. Studies have indicated that weight loss is not secondary to inadequate nutrition, nor to hyperactivity^5^. Studies have instead suggested that loss of body weight results from changes in metabolism^7^ and also that reduced absorption of nutrients along the intestinal tract may play a role^8^. Work mostly performed in HD mouse models has demonstrated that tissues and organs that are involved in nutrient absorption are affected^8^.

In HD mouse models, huntingtin aggregates are abundantly present along the gastrointestinal tract^9^. The R6/2 mouse, the most widely studied transgenic animal model of HD, exhibits loss of enteric neuropeptides and altered gut motility^8^. Gastrointestinal function has never been investigated in HD patients, but there are indications that it may be affected. Patients are prone to suffer from gastritis and esophagitis^10^.

We therefore set out to study the gastric mucosa, using gastric mucosal biopsies as a tool, to look for abnormalities of enteric neurons and mucosal cells.

## Materials and methods

Patient demographics

Patients with HD lose weight and have feeding difficulties. In some cases, this is managed by the insertion of a percutaneous endoscopic gastrostomy (PEG) feeding tube. Ethical approval (MREC No. 08/WSE02/66) was given to approach patients after a clinical decision to insert a PEG. Gastric biopsies (from antrum and fundus/gastric body) were obtained from twelve HD subjects during the procedure to insert the PEG. Using the total functional capacity (TFC) rating scale^11^: 9 patients were at stage 5 (TFC = 0), one patient was at stage 4 (TFC = 1-2) and one patient was at stage 2 of the disease (TFC = 7-10) and had a TFC of 7. The patients were in long-term care and the formal CAG length report was not available for 8 patients (Table 1).

Control samples were obtained from 10 patients; 9 were being investigated for possible coeliac disease, one for altered bowel habit; the gastric mucosa was considered normal by the endoscopist. Ethical approval, covering England and Wales, was granted by the South East Wales Research Ethics Committee (08/WSE02/66) and confirmed in Scotland by the Scottish A Research Ethics Committee (08/MRE00/85). Written informed consent was obtained from all participants in this study.

**Table 1:** Patient demographics

Group	N (M/F)	Mean Age (Range)
Control	10 (8/2)	55.5 (41-71)
HD	12 (6/6)	55.8 (25-73)

Immunohistochemistry

The gastric biopsies were fixed in formaldehyde and embedded in paraffin wax according to routine procedures.

Antrum and fundus (gastric body) were cut into 7 μm thick sections using a microtome (Leica SM2010R, Leica Biosystems Nussloch GmbH, Nussloch, Germany).

The different cell types were identified using immunohistochemistry; antrum sections – D-cells (anti-somatostatin antibody raised in rabbit; 1:3000 dilution, kind gift from Prof. J.J. Holst, Copenhagen University, Denmark), G cells (anti-gastrin; 1:2000 dilution raised in rabbit, kind gift from Prof. J.E. Rehfeld, Copenhagen University, Denmark) and fundus (gastric body) sections – parietal cells (anti-H+/K+ ATPase antibody raised in mouse; 1:1000 dilution, kind gift from Prof. A.J. Smolka, UCLA, USA), chief cells (anti-pepsinogen antibody raised in swine, 1:1000 dilution, kind gift from Prof. P.T. Sangild, Copenhagen University, Denmark), endocrine cells (polyclonal anti-chromogranin A raised in goat; 1:1000 dilution, Santa Cruz Biotechnology Inc., Santa Cruz, CA, USA). Antibodies were diluted in PBS containing 0.25% Triton X-100 and 0.25% bovine serum albumin. Prior to immunostaining, sections underwent antigen retrieval by boiling in citrate buffer using a microwave. Sections were incubated with primary antibodies overnight at 4°C in the dark in a humid chamber. The next day, sections were incubated with the appropriate secondary antibodies for 1h at room temperature, followed by DAPI (1:2000, Sigma-Aldrich, Stockholm, Sweden) for 10 minutes: DyLightTM 488-conjugated AffiniPure donkey anti-mouse, 1:1000, Jackson ImmunoResearch Laboratories Inc., PA, USA; FITC-conjugated AffiniPure goat anti-swine, 1:100, BioNordika, Stockholm, Sweden; Cy2-conjugated AffiniPure donkey anti-rabbit, 1:300, Jackson ImmunoResearch; Cy2-conjugated AffiniPure donkey anti-goat, 1:500, Jackson ImmunoResearch. Control incubations were also included without the use of primary antibody; no staining was observed in these sections.

Immunofluorescence was examined using an epi-fluorescence microscope (Olympus BX53, Olympus, Tokyo, Japan) and digital images were acquired using a digital camera (Olympus DP73, Olympus, Tokyo, Japan). Section areas in the antrum with immunostaining against G cells were measured in digitized images using cellSens Dimensions 1.11 software (Olympus, Tokyo, Japan).

Cells were counted upon staining and expressed as total number of positive cells within the whole section (G cells) and related to area of section, or total number of cells per visual field (parietal cells, chief cells and endocrine cells, 400 μm^2^ and D cells, 100 μm^2^).

Statistical analysis

All data were analysed using GraphPad Prism 6 (GraphPad Software Inc., San Diego, CA, USA). Data are presented as mean ± SEM, with p < 0.05, one-tailed t-test considered as statistically significant.

## Results and discussion

Autolysis prevents the use of human post mortem tissue, therefore in this study, gastric biopsies were obtained upon a clinical decision to insert a feeding tube. Control samples were obtained from patients being investigated for a possible diagnosis of coeliac disease, however, these patients were not considered to have any gastric abnormality. This group of controls were chosen because it was unlikely that they would have any neurodegenerative disease, although one patient had cerebellar ataxia and was being investigated for a possible gluten enteropathy; his problems were eventually considered to be due to an excess intake of alcohol. One of the control patients was being investigated for altered bowel habit and found to have an oesophageal adenocarcinoma arising from a Barrett’s mucosa. Only one patient was established to have coeliac disease.

In order to investigate possible histological differences related to gastric function, we used immunohistochemistry to evaluate the expression of cell specific markers of 2 exocrine cell types in fundus (gastric body) sections, acid-producing parietal cells and pepsinogen producing chief cells as well as markers of 2 endocrine cell types in antrum sections, gastrin producing cells and somatostatin producing cells. We also stained fundus (gastric body) sections with chromogranin A, a protein found in secretory vesicles of endocrine cells and neurons.

In line with previous HD mouse studies showing reduction of GI tract neuropeptides^8^, using immunohistochemistry, we detected a reduction in the cell density of G-cells in antrum biopsies from HD subjects compared to the control group (Figure 1). We also observed an increase in the cell density of pepsinogen-producing chief cells of the fundus (gastric body) (Figure 1). Possibly, the latter could play a role in the increased risk of gastritis/esophagitis in HD6 since increased levels of pepsin (the active form of pepsinogen) are associated with formation of peptic ulcers^12^^,^ 13 ^,^ 14. There was no change seen in the cell density of gastric acid producing parietal cells. Similarly, there was no change in the cell density of somatostatin-producing D cells, nor in the total number of endocrine cells (as revealed by their chromogranin A expression) in the fundus (gastric body) (Table 2). Interestingly, gastrin is the hormone that upon food intake evokes acid secretion from the parietal cell, mediated by the subsequent histamine release from the ECL cell, indicating that there might possibly be an alteration in parietal cell stimulation in HD subjects.

**Representative images and cell counts:**
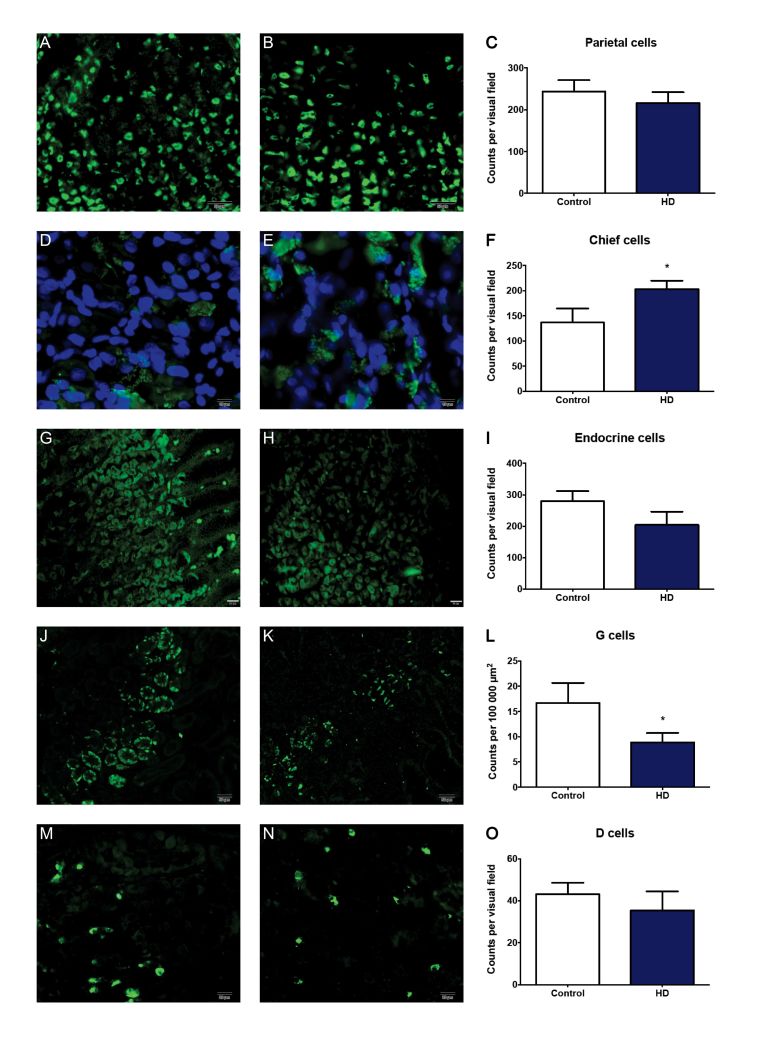
**A, B, D, E, G, H, J, K, M** and **N** show representative fluorescence microscope images of human stomach sections stained for various cell types. **A, D, G, J** and **M** are from control subjects, while **B, E, H, K** and **N **are from HD patients. Panels **C, F, I, L** and **O** show cell counts for HD patients vs. control as indicated. Bars represent mean ± SEM. Scale bars: **A, B, J, K** – 50 µm; **G, H, M, N** – 20 µm; **D, E** – 10 µm. Visual field areas analysed were 400 μm2 for parietal cells, chief cells and endocrine cells, and 100 μm2 for D cells. No significant differences were found between HD patients and controls in fundus (gastric body) sections stained for the presence of parietal cells (**A, B** and **C**) or endocrine cells (**G, H** and **I**), nor antrum sections for the presence of D cells (**M, N** and **O**). Significant differences were observed in fundus (gastric body) sections stained for chief cells (**D, E** and **F**), where HD patients had a greater cell density of positive cells, and antrum sections stained for gastrin producing G cells (**J, K** and **L**), with HD patients having fewer counts than controls.

**Table 2:** Cell counts

Cell type	Stomach region	Group	Mean ± SEM	N	P value
Parietal cells	Fundus (gastric body)	Control	243.9 ± 26.78	9	
		HD	215.7 ± 26.33	10	> 0.05
Chief cells	Fundus (gastric body)	Control	136.8 ± 28.14	9	
		HD	203.2 ± 16.79	9	0.0298
Endocrine cells	Fundus (gastric body)	Control	279.0 ± 32.32	6	
		HD	204.2 ± 41.88	5	> 0.05
G cells	Antrum	Control	16.68 ± 3.994	8	
		HD	8.884 ± 1.845	8	0.0490
D cells	Antrum	Control	43.10 ± 5.496	10	
		HD	35.43 ± 9.068	7	> 0.05

In summary, our results indicate that in late stage HD, alterations in mucosal cells exist, however, further studies are needed in order to evaluate whether these alterations lead to functional consequences.
